# Commentary: Mindfulness and CBT: a conceptual integration bridging ancient wisdom and modern cognitive theories of psychopathology

**DOI:** 10.3389/fpsyg.2025.1565835

**Published:** 2025-04-22

**Authors:** Aldo Aguirre-Camacho

**Affiliations:** Department of Psychology, Faculty of Biomedical and Health Sciences, Universidad Europea de Madrid, Villaviciosa de Odón, Spain

**Keywords:** mindfulness, CBT, ACT, functional contextualism, philosophical assumptions, psychotherapy

## 1 Introduction

I read Beshai's (2024) conceptual analysis on mindfulness and cognitive behavioral therapy (CBT) with interest. The author seeks to “illustrate how mindfulness, as a third-wave approach, can complement and enhance first- and second-wave approaches.”

The so-called cognitive behavioral therapies comprise a variety of therapeutic approaches typically categorized into three waves: first (e.g., behavior therapy and behavior modification), second (e.g., rational emotive behavior therapy and CBT), and third (e.g., acceptance and commitment therapy (ACT), dialectical behavioral therapy, and functional analytic psychotherapy) (Hayes, 2004). Despite broad similarities, such as shared techniques/strategies and a focus on the present, cognitive behavioral therapies are underpinned by different philosophical assumptions (O'Donohue and Chin, 2022). Herein, I discuss theoretical inconsistencies pertaining to the implementation of mindfulness within Beckian CBT. I illustrate these inconsistencies by comparing CBT and ACT— the therapeutic approaches that have arguably received the most attention within their respective waves—while relying on a secular view of mindfulness as defined by Kabat-Zinn (2003): “paying attention on purpose, in the present moment, and non-judgementally to the unfolding of experience moment by moment.”

## 2 Philosophical assumptions

CBT is based on Stoicism (Beck et al., 1979), a pre-Socratic philosophy emphasizing the rational control of thought and emotion. The influence of Stoicism is evident in CBT's emphasis on teaching clients to critically assess and modify the content of dysfunctional thoughts/beliefs to positively impact emotion and behavior (Dobson and Dozois, 2001). Such an emphasis, along with CBT's reliance on Kant's (1781/1929) concept of “schemas” (Dozois and Beck, 2011), reveals an ontological worldview based on elemental realism (i.e., mechanism) and mind–body dualism, as well as a focus on content rather than function (O'Donohue and Chin, 2022). These aspects highlight CBT's detachment from the behavioral and contextual roots of first- and third-wave approaches.

ACT is based on functional contextualism, a scientific philosophy rooted in Skinner's radical behaviorism and first-wave behavior analytical principles (O'Donohue and Chin, 2022). Functional contextualism assumes a monistic stance, wherein private events (i.e., thinking, feeling, paying attention) and overt behavior are ontologically alike. Therapeutic work within ACT generally focuses on the function of behavior—either private or overt—within its context. The morphology of behavior (e.g., thought content) is not usually of primary importance (Hayes et al., 2012).

## 3 Discussion

The worldviews underlying CBT and ACT constitute divergent perspectives on human behavior and, as a result, should orient psychotherapy in different directions (Marica, 2015). This may manifest in dissimilar views on what may count as clinically relevant behavior and mechanisms of change, ultimately leading to different case formulations. More pertinent to this discussion, such divergent worldviews should result in different conceptualizations of mindfulness and its so-called mechanisms of action, which may be reflected in the way mindfulness is integrated into clinical practice.

Mindfulness is almost synonymous with third-wave approaches. The term “mindfulness” is often presented within the ACT literature as a *middle-level term* (i.e., a non-technical term) (O'Donohue and Chin, 2022). Nonetheless, from a functional contextualist perspective, mindfulness can be largely conceptualized as a set of behaviors or skills to be learned (e.g., paying attention, observing rather than judging) (Kohlenberg et al., 2009). As a therapeutic resource, mindfulness is also congruent with therapeutic targets within ACT. For example, the implementation of mindfulness seeks to change the way clients relate to thoughts/emotions without necessarily modifying them. In behavioral analytic terms, it seeks to alter the eliciting and/or evoking functions of private events that may narrow an individual's behavioral repertoire and/or interfere with valued action (Hayes et al., 2012; Wilson et al., 2012). Participation in mindfulness-based interventions has been associated with a myriad of benefits (e.g., reduced psychopathology, emotion regulation, and positive reappraisal), which have been attributed to a small set of mechanisms of change (or learned skills), such as decentering (Garland et al., 2015; Grabovac et al., 2011; Shapiro et al., 2006).

I contend that the implementation of mindfulness within CBT conflicts with the philosophical assumptions underlying CBT. First, as noted by Beshai (2024) and others (Beck, 1970), CBT's philosophical assumptions postulate that change in thought content is essential for change in behavior and emotion. CBT clients are routinely taught to critically assess their beliefs to identify potential maladaptive cognitions; when distancing from thoughts is promoted, it is done to change thought content (Dozois and Beck, 2011). As discussed above, however, mindfulness is implemented within ACT on the assumption that private events (e.g., thoughts) do not need to be altered for change in emotion or behavior to occur (Germer et al., 2016). Rather, the practice of mindfulness is meant to promote decentering from private events, which often leads to reduced suffering and facilitate desired behavior change (Germer et al., 2016). Second, CBT embraces a content-oriented view of the self, believed to be comprised of cognitive structures (e.g., core beliefs). In contrast, the Buddhist philosophy underlying mindfulness contends that the self is an illusion (i.e., *anatta*) (Germer et al., 2016); this is consistent with functional contextualism's (and radical behaviorism's) view of the self, conceptualized as a behavioral repertoire rather than an individual entity (Wilson et al., 2012).

In summary, the ultimate question posed here is whether mindfulness can be assimilated within CBT in a way that is consistent with CBT's underlying philosophical assumptions, that is, whether “theoretically consistent eclecticism” is possible (Dryden, 1987). I don't think it is, given that what mindfulness is (Germer et al., 2016) seems to challenge CBT's fundamental propositions. Therefore, although cognitive behavior therapies share a variety of techniques and strategies, I do not think “technical eclecticism” is possible in this case (Lazarus, 1995).
